# Perceptions towards charcoal-burning suicide and the surge of this lethal method in Taiwan

**DOI:** 10.1371/journal.pone.0262384

**Published:** 2022-01-21

**Authors:** Yi-Ju Pan, Mei-Xian Loi, Yin-Hsiang Lan, Chun-Lin Chen, I-Chih Cheng

**Affiliations:** 1 Department of Psychiatry, Far Eastern Memorial Hospital, New Taipei City, Taiwan; 2 Institute of Public Health, School of Medicine, National Yang Ming Chiao Tung University, Taipei, Taiwan; 3 Center for General Education, Taipei Medical University, Taipei, Taiwan; Columbia University, UNITED STATES

## Abstract

**Objective:**

Whether sociocultural perceptions of charcoal-burning suicide have influenced its rapid increase in prevalence is unclear. We aimed to explore perceptions of Taiwan’s general population regarding charcoal-burning suicide, their personal belief in life after death, and related feelings of thoughts associated with those who attempt charcoal-burning suicide.

**Methods:**

An online web-based survey, focussing on sociocultural attitudes towards death, as well as perceptions towards charcoal-burning suicide, and those who attempt charcoal-burning suicide, was conducted from 14 January to 14 June 2016.

**Results:**

In total, 1343 adults completed the online survey (mean age of 33.46; 66.6% women). Notably, 90.3% of participants considered charcoal burning to be an easily accessible suicide method. Multivariable analyses revealed that among the examined factors, the perceived ‘painlessness’ of charcoal-burning suicide was associated with an over seven-fold increased risk of choosing charcoal-burning suicide (OR = 7.394; p < 0.001; 95% CI: 2.614–20.912).

**Conclusion:**

As reflected in this study, charcoal-burning suicide is perceived as easily accessible and painless. The perceived ‘painlessness’ may be the factor that distinguishes the choice of charcoal-burning suicide from that of other suicide methods. Future efforts to target these perceptions regarding charcoal-burning suicide may be warranted in both media reporting and suicide prevention programmes.

## Introduction

The first report of charcoal burning as a suicide method in Hong Kong in November 1998 resulted in a suicide epidemic in the local area, and charcoal burning quickly evolved into a contagious suicide method in the Chinese communities of Macau in coastal southern China and in Taiwan [1, 2]. A charcoal-burning suicide epidemic also occurred in Japan from 2003 to 2007 [3] and subsequently in Korea from 2007 to 2011 [4, 5]. During these epidemics, charcoal burning became a leading suicide method in Taiwan, particularly among the younger population [6]. In Japan, the charcoal-burning suicide epidemic led to an increase of more than 10% in the overall suicide rate among individuals aged 25–44 years, without an apparent decrease in other methods of suicide [3], which indicated the possibility that charcoal burning appealed to many individuals who might not have used other highly lethal methods if charcoal burning had not been an accessible method.

Although preferred suicide methods vary by country, the primary suicide method typically changes slowly. Charcoal burning, however, is an exception, according to a report based on the World Health Organization mortality database [7]. The exceptionally rapid adoption of a new suicide method (i.e., charcoal burning) suggested the presence and critical role of cultural preparedness in susceptible societies. Cultural attitudes towards death can influence suicide method decisions [8]. The geographical distribution of charcoal-burning suicide epidemics, which coincides with the distribution of closely related Eastern Asian cultures, implies the potential role of sociocultural factors in this epidemic. Apart from the general sociocultural factors, the public’s perception of charcoal-burning suicide may influence the preference for or adoption of this lethal method. A report posited that the media implicitly conveys the message that charcoal burning is ‘an easily accessible, effective, and painless means of killing oneself’ [9]. Furthermore, an interview-based study of suicide survivors in Taiwan revealed that individuals who had attempted charcoal-burning suicide were more likely than those who had attempted other methods of suicide to report that their choice of method was influenced by the media, particularly the portrayal of charcoal burning as ‘a peaceful way of dying’ [10]. Few studies have specifically researched into sociocultural perceptions of charcoal-burning suicide and their potential impacts.

Previous studies addressing the relationships between religiosity and suicidal behaviours have yielded inconclusive results [11, 12]. Contrarily, a study of the characteristics of suicide notes in China revealed that 25.1% of note leavers mentioned life after death, for example, they could be reborn and have another life, they would bless their living family members after death, or they would become wild ghosts. Accordingly, the researchers suggested that rather than religious beliefs, the belief of an afterlife is likely to be related to suicide risk [13].

The influence of the media on the spread of charcoal-burning suicide has not been fully explored [14, 15]. In Japan, a suicide pact among young strangers who had met online and died of suicide through charcoal burning attracted considerable media attention and led to the formation of copycat suicide pacts [16–18]. A South Korean study suggested that Internet reports of suicide through charcoal burning tend to precede an increased incidence of suicide with this method. Moreover, the researchers believed that one episode of heavy media coverage of a novel suicide method, such as charcoal burning, is sufficient to increase the prevalence of suicide by that method even after media coverage has decreased [5]. In a previous Hong Kong study that involved in-depth ethnographic interviews with individuals who had survived serious suicide attempts through charcoal burning, nearly all survivors claimed that they learned of and were reminded of this suicide method through the media [14]. Research clarifying the influence of the media on the perceptions of charcoal-burning suicide and those who attempt suicide by this method remains relatively lacking.

Examining the features of cultural preparedness and general perceptions of charcoal-burning suicide may be key to understanding the determining factors for the adoption of a new suicide method in susceptible communities in the future. Therefore, in this online survey study, we explored whether sociocultural attitudes towards life after death, perceptions towards charcoal-burning suicide and the role of media on it, as well as those who attempt suicide by charcoal burning have influenced the adoption of this suicide method.

## Materials and methods

### Ethical standards

The authors of this study asserted that all procedures contributing to this work comply with the ethical standards of the relevant national and institutional committees on human experimentation and with the Helsinki Declaration of 1975, as revised in 2008. This survey was approved by the research ethics review committee of Far Eastern Memorial Hospital of Taiwan (104118-F).

### Study sample

In this study, we recruited participants aged 20 years or older through an online web-based survey, focussing on sociocultural perceptions towards death, suicide, charcoal-burning suicide, and those who attempt suicide by charcoal burning, in Taiwan. This survey was conducted on a professional online web-based survey platform (MySurvey, http://www.mysurvey.tw/index.htm), which ensured the safety and security of our confidential data. A link to the web-based questionnaire was distributed on Facebook and various social media platforms and fora to obtain the most heterogeneous sample possible. The survey period was 5 months, between 14 January and 14 June 2016. Through these Internet sources, we recruited 1343 participants (66.6% female; mean age 33.46 years, SD 9.47) who responded to and completed this online survey without any missing responses. Table 1 provides descriptive statistics of the sample demographics.

**Table 1:** Demographic data, psychiatric symptoms during lifetime, and conceptualisation of life after death in relation to presence of suicide ideation.

	Participants with suicide ideation, *n* = 410	Participants without suicide ideation, *n* = 933	Total, *N* = 1343	Significance
Age [mean (SD)]	32.15(9.130)	34.04(9.567)	33.46(9.472)	F = 11.379, *p* = 0.001*
Sex [n (%)]				χ^2^ = 6.812, *p* = 0.009*
Male	116(28.3)	332(35.6)	448(33.4)	
Female	294(71.7)	601(64.4)	895(66.6)	
Education level [n (%)]				χ^2^ = 1.874, *p* = 0.392
Master’s degree	94(22.9)	195(20.9)	289(21.5)	
Bachelor’s degree	275(67.1)	623(66.8)	898(66.9)	
High school or lower	41(10.0)	115(12.3)	156(11.6)	
Marital status [n (%)]				χ^2^ = 20.012, *p* = 4.5x10^-5^**
Unmarried	271(66.1)	518(55.5)	789(58.7)	
Married	124(30.2)	397(42.6)	521(38.8)	
Other	15(3.7)	18(1.9)	33(2.5)	
Frequency of Internet usage [n (%)]				χ^2^ = 2.566, *p* = 0.277
Almost always online	343(83.7)	767(82.2)	1110(82.7)	
Online at least once per day	63(15.4)	145(15.5)	208(15.5)	
Online less than once per day	4(1.0)	21(2.3)	25(1.9)	
History of psychiatric outpatient clinic visit [n (%)]				χ^2^ = 63.664, *p* = 1.5x10^-15^**
Yes	64(15.6)	32(3.4)	96(7.1)	
No	346(84.4)	901(96.6)	1247(92.9)	
Symptoms of depression [n (%)]				χ^2^ = 42.442, *p* = 7.3x10^-11^**
Yes	42(10.2)	20(2.1)	62(4.6)	
No	368(89.8)	913(97.9)	1281(95.4)	
Symptoms of insomnia [n (%)]				χ^2^ = 9.754, *p* = 0.002*
Yes	35(8.5)	40(4.3)	75(5.6)	
No	375(91.5)	893(95.7)	1268(94.4)	
Symptoms of anxiety or other psychiatric diagnosis [n (%)]				χ^2^ = 9.154, *p* = 0.002*
Yes	35(8.5)	41(4.4)	76(5.7)	
No	375(91.5)	892(95.6)	1267(94.3)	
History of attempted suicide [n (%)]				χ^2^ = 146.977, *p* = 8.0x10^-34^**
Yes	65(15.9)	2(0.2)	67(5.0)	
No	345(84.1)	931(99.8)	1276(95.0)	
Belief that people’s souls go to heaven after death [n (%)]				χ^2^ = 0.163, *p* = 0.687
Yes	119(29.0)	281(30.1)	400(29.8)	
No	291(71.0)	652(69.9)	943(70.2)	
Belief that people’s souls go to hell after death [n (%)]				χ^2^ = 0.430, *p* = 0.512
Yes	105(25.6)	255(27.3)	360(26.8)	
No	305(74.4)	678(72.7)	983(73.2)	
Belief that people’s souls remain in the world after death [n (%)]				χ^2^ = 0.419, *p* = 0.518
Yes	84(20.5)	177(19.0)	261(19.4)	
No	326(79.5)	756(81.0)	1082(80.6)	
Belief that people enter the cycle of reincarnation after death [n (%)]				χ^2^ = 0.267, *p* = 0.605
Yes	115(28.0)	249(26.7)	364(27.1)	
No	295(72.0)	684(73.3)	979(72.9)	
Belief that people repeat the dying process after death [n (%)]				χ^2^ = 0.258, *p* = 0.612
Yes	84(20.5)	180(19.3)	264(19.7)	
No	326(79.5)	753(80.7)	1079(80.3)	
Belief that people’s souls remain with loved ones after death [n (%)]				χ^2^ = 6.369, *p* = 0.012*
Yes	109(26.6)	190(20.4)	299(22.3)	
No	301(73.4)	743(79.6)	1044(77.7)	
Belief that people’s souls disappear after death [n (%)]				χ^2^ = 13.998, *p* = 1.8x10^-4^**
Yes	172(42.0)	293(31.4)	465(34.6)	
No	238(58.0)	640(68.6)	878(65.4)	
Uncertainty regarding where people’s souls go after death [n (%)]				χ^2^ = 1.702, *p* = 0.192
Yes	66(16.1)	178(19.1)	244(18.2)	
No	344(83.9)	755(80.9)	1099(81.8)	

The ‘High school or lower ‘category under ‘Education level’ includes those in high school, elementary school, and with no education.

The ‘Other’ category under ‘Marital status’ includes those separated, divorced, or widowed. Other psychiatric diagnoses include panic disorder, obsessive-compulsive disorder, schizophrenia, bipolar disorder, and any other psychiatric disorder.

*p < 0.05

**p < 0.001.

### Measures

We used a structured questionnaire which included three main sections. The questionnaire was developed purely for the current study to explore potential presence of beliefs in life after death, presence of suicide ideation, perceptions towards charcoal burning and perceptions towards those who attempted suicide by charcoal burning. The language used in the questionnaire is Chinese. All participants gave their consents before answering the survey questions. The first part of the questionnaire included questions regarding basic information, such as age, gender, education level (master’s degree, bachelor’s degree, high school, elementary school, no education), marital status (unmarried, married, separated, divorced, widowed), estimated frequency of Internet usage (almost always online, online at least once per day, online less than once per day), history of psychiatric outpatient clinic visits, lifetime experiences of psychiatric symptoms or diagnoses, history of suicide ideation, incidences of attempted suicide, and suicide ideation of charcoal burning. The second part of the questionnaire comprised multiple choice questions regarding personal beliefs or conceptualisations of death and life after death, such as ‘Belief that people’s spirits or souls go to heaven after they die’ ‘Belief that people’s spirits or souls disappear after they die’ ‘Belief that people’s spirits or souls enter the cycle of reincarnation after they die’ and ‘Not sure about where people’s spirits or souls go after they die’. The participant can choose as many answers as possible and we presented ‘Yes’ in Table 1 when the participant chose the answer and vice versa. The third part comprised questions regarding personal perceptions of individuals who choose charcoal burning as their suicide method and personal perceptions of charcoal-burning suicide, such as ‘What kind of person are those who choose charcoal burning as their suicide method? A. Those who are accustomed to watching the news; B. Those who are not accustomed to watching the news; C. Not Applicable’ and ‘How do you feel about charcoal-burning suicide? Is it A. Painful; B. Painless; C. Not Applicable’.

### Statistical analysis

Demographic data (age, gender, education level, marital status, and frequency of Internet usage), history of psychiatric illness, visits to psychiatric outpatient clinics, and suicide ideation, suicide attempts, as well as suicide ideation of charcoal burning during their lifetimes were described in the overall sample and compared between groups according to presence of suicide ideation and ideation of charcoal-burning suicide. Age as a continuous variable was compared by two-sample t-test and categorical variables were compared by chi-squared test. To further explore the nature of the relationships between sociocultural perceptions and charcoal-burning suicide, separate binary logistic regression analyses were conducted to estimate the differential associations of demographic data, history of psychiatric illnesses, conceptualisation of death or life after death, perceptions of charcoal-burning suicide, as well as perceptions towards those who attempt suicide by charcoal burning, with presence of suicide ideation, and presence of charcoal-burning suicide ideation as outcome variables, respectively. The detailed exposure variables for logistic regressions were presented in the Tables. Sensitivity analysis was conducted to predict ideation of charcoal-burning suicide among individuals who had contemplated suicide. All statistical analyses were performed with SPSS (version 20.0; IBM, Armonk, NY, USA), and the significance level was set at 0.05 (two-tailed) with a 95% confidence interval.

## Results

In total, 1343 adults aged older than 20 years (age range: from 20 to 75 years old; standard deviation: 9.47) completed the online survey. Their sociodemographic characteristics are presented in Table 1. The mean age of this sampled population was 33.46 years, and the majority were women (66.6%). Among individuals who completed the survey, 82.7% considered themselves to be frequent Internet users. According to their reported subjective experiences, 12.1% of them had suffered from psychiatric symptoms or mental illnesses, including depression, anxiety, sleep disturbance, panic attacks, obsessive-compulsive disorder, bipolar disorder, or schizophrenia during their lifetime. Of all study participants, 7.1% reported that they had psychiatric outpatient visits. Approximately 30% (n = 410) of those surveyed reported having had suicidal thoughts during their lifetime, and 5% (n = 67) reported having attempted suicide. Of all surveyed participants, 4.3% (n = 58) had considered suicide through charcoal burning themselves (Table 2). In multivariable logistic regressions, we observed that younger age, female sex, a history of psychiatric outpatient visits, a history of attempted suicide, and belief that the spirit remains with loved ones or disappears after death were associated with higher risk of suicidal ideation (Table 3).

**Table 2:** Demographic data, psychiatric symptoms during lifetime, conceptualisation of life after death, and perceptions of charcoal-burning suicide in relation to presence of charcoal-burning suicide ideation.

	Participants with charcoal-burning ideation, *n* = 58	Participants without charcoal-burning ideation, *n* = 1285	Total, *N* = 1343	Significance
Age [mean (SD)]	32.03(9.683)	33.53(9.462)	33.46(9.472)	F = 1.381, *p* = 0.240
Sex [n (%)]				χ^2^ = 0.221, *p* = 0.638
Male	21(36.2)	427(33.2)	448(33.4)	
Female	37(63.8)	858(66.8)	895(66.6)	
Education level [n (%)]				χ^2^ = 0.659, *p* = 0.719
Master’s degree	10(17.2)	279(21.7)	289(21.5)	
Bachelor’s degree	41(70.7)	857(66.7)	898(66.9)	
High school or lower	7(12.1)	149(11.6)	156(11.6)	
Marital status [n (%)]				χ^2^ = 11.597, *p* = 0.003*
Unmarried	37(63.8)	752(58.5)	789(58.7)	
Married	16(27.6)	505(39.3)	521(38.8)	
Other	5(8.6)	28(2.2)	33(2.5)	
Frequency of Internet usage [n (%)]				χ^2^ = 1.790, *p* = 0.409
Almost always online	51(87.9)	1059(82.4)	1110(82.7)	
Online at least once per day	7(12.1)	201(15.6)	208(15.5)	
Online less than once per day	0(0.0)	25(1.9)	25(1.9)	
History of psychiatric outpatient clinic visits [n (%)]				χ^2^ = 21.284, *p* = 4.0x10^-6^**
Yes	13(22.4)	83(6.5)	96(7.1)	
No	45(77.6)	1202(93.5)	1247(92.9)	
Symptoms of depression [n (%)]				χ^2^ = 43.603, *p* = 4.0x10^-11^**
Yes	13(22.4)	49(3.8)	62(4.6)	
No	45(77.6)	1236(96.2)	1281(95.4)	
Symptoms of insomnia [n (%)]				χ^2^ = 2.605, *p* = 0.107
Yes	6(10.3)	69(5.4)	75(5.6)	
No	52(89.7)	1216(94.6)	1268(94.4)	
Symptoms of anxiety or other psychiatric diagnosis [n (%)]				χ^2^ = 2.493, *p* = 0.114
Yes	6(10.3)	70(5.4)	76(5.7)	
No	52(89.7)	1215(94.6)	1267(94.3)	
History of attempted suicide [n (%)]				χ^2^ = 38.830, *p* = 4.6x10^-10^**
Yes	13(22.4)	54(4.2)	67(5.0)	
No	45(77.6)	1231(95.8)	1276(95.0)	
Belief that people’s souls go to heaven after death [n (%)]				χ^2^ = 0.256, *p* = 0.613
Yes	19(32.8)	381(29.6)	400(29.8)	
No	39(67.2)	904(70.4)	943(70.2)	
Belief that people’s souls go to hell after death [n (%)]				χ^2^ = 0.553, *p* = 0.457
Yes	18(31.0)	342(26.6)	360(26.8)	
No	40(69.0)	943(73.4)	983(73.2)	
Belief that people’s souls remain in the world after death [n (%)]				χ^2^ = 1.600, *p* = 0.206
Yes	15(25.9)	246(19.1)	261(19.4)	
No	43(74.1)	1039(80.9)	1082(80.6)	
Belief that people enter the cycle of reincarnation after death [n (%)]				χ^2^ = 0.149, *p* = 0.699
Yes	17(29.3)	347(27.0)	364(27.1)	
No	41(70.7)	938(73.0)	979(72.9)	
Belief that people repeat the dying process after death [n (%)]				χ^2^ = 0.018, *p* = 0.892
Yes	11(19.0)	253(19.7)	264(19.7)	
No	47(81.0)	1032(80.3)	1079(80.3)	
Belief that people’s souls remain with loved ones after death [n (%)]				χ^2^ = 5.230, *p* = 0.022*
Yes	20(34.5)	279(21.7)	299(22.3)	
No	38(65.5)	1006(78.3)	1044(77.7)	
Belief that people’s souls disappear after death [n (%)]				χ^2^ = 0.067, *p* = 0.796
Yes	21(36.2)	444(34.6)	465(34.6)	
No	37(63.8)	841(65.4)	878(65.4)	
Uncertainty regarding where people’s souls go after death [n (%)]				χ^2^ = 0.287, *p* = 0.592
Yes	9(15.5)	235(18.3)	244(18.2)	
No	49(84.5)	1050(81.7)	1099(81.8)	
Do you think that people who choose charcoal burning for suicide are accustomed to surfing the Internet? [n (%)]				χ^2^ = 6.209, *p* = 0.013*
Yes	38(65.5)	627(48.8)	665(49.5)	
No or uncertain	20(34.5)	658(51.2)	678(50.5)	
Do you think that people who choose charcoal burning for suicide are accustomed to watching the news? [n (%)]				χ^2^ = 6.527, *p* = 0.011*
Yes	46(79.3)	807(62.8)	853(63.5)	
No or uncertain	12(20.7)	478(37.2)	490(36.5)	
Do you think that people who choose charcoal burning for suicide live alone? [n (%)]				χ^2^ = 0.023, *p* = 0.880
Yes	33(56.9)	744(57.9)	777(57.9)	
No or uncertain	25(43.1)	541(42.1)	566(42.1)	
Do you think that people who choose charcoal burning for suicide are poor? [n (%)]				χ^2^ = 0.133, *p* = 0.715
Yes	34(58.6)	784(61.0)	818(60.9)	
No or uncertain	24(41.4)	501(39.0)	525(39.1)	
Do you think that people who choose charcoal burning for suicide are educated? [n (%)]				χ^2^ = 7.207, *p* = 0.007*
Yes	35(60.3)	546(42.5)	581(43.3)	
No or uncertain	23(39.7)	739(57.5)	762(56.7)	
Do you think that charcoal-burning suicide is painless? [n (%)]				χ^2^ = 36.754, *p* = 1.3x10^-9^**
Yes	53(91.4)	652(50.7)	705(52.5)	
No or uncertain	5(8.6)	633(49.3)	638(47.5)	
Do you think that charcoal-burning suicide keeps the body intact? [n (%)]				χ^2^ = 17.139, *p* = 3.5x10^-5^**
Yes	45(77.6)	640(49.8)	685(51.0)	
No or uncertain	13(22.4)	645(50.2)	658(49.0)	
Do you think that charcoal-burning suicide involves suffocation? [n (%)]				χ^2^ = 2.193, *p* = 0.139
Yes	27(46.6)	725(56.4)	752(56.0)	
No or uncertain	31(53.4)	560(43.6)	591(44.0)	
Do you think that charcoal-burning suicide is beautiful? [n (%)]				χ^2^ = 31.216, *p* = 2.3x10^-8^**
Yes	29(50.0)	251(19.5)	280(20.8)	
No or uncertain	29(50.0)	1034(80.5)	1063(1063)	
Do you think that charcoal is easily accessible? [n (%)]				χ^2^ = 0.537, *p* = 0.464
Yes	54(93.1)	1159(90.2)	1213(90.3)	
No or uncertain	4(6.9)	126(9.8)	130(9.7)	
Do you believe that charcoal-burning suicide leads to burns or amputation? [n (%)]				χ^2^ = 0.491, *p* = 0.484
Yes	20(34.5)	502(39.1)	522(38.9)	
No or uncertain	38(65.5)	783(60.9)	821(61.1)	
Do you believe that charcoal-burning suicide leads to lung injury? [n (%)]				χ^2^ = 0.068, *p* = 0.795
Yes	53(91.4)	1161(90.4)	1214(90.4)	
No or uncertain	5(8.6)	124(9.6)	129(9.6)	
Do you believe that charcoal-burning suicide leads to delayed brain injury? [n (%)]				χ^2^ = 0.567, *p* = 0.452
Yes	46(79.3)	963(74.9)	1009(75.1)	
No or uncertain	12(20.7)	322(25.1)	334(24.9)	

The ‘High school or lower’ category under ‘Education level’ includes those in high school, elementary school, and with no education.

The ‘Other’ category under ‘Marital status’ includes those separated, divorced, and widowed. Other psychiatric diagnoses include panic disorder, obsessive-compulsive disorder, schizophrenia, bipolar disorder, and any other psychiatric disorder.

*p < 0.05

**p < 0.001.

**Table 3:** Odds ratios and 95% confidence intervals for the risk of having suicide ideation (N = 1,343).

			95% CI for OR	
	Significance	OR	Lower	Upper
History of psychiatric outpatient clinic visits				
Yes vs. No	1.8x10^-5^**	3.343	1.925	5.805
History of attempted suicide				
Yes vs. No	8.0x10^-10^**	92.507	21.831	391.997
Belief that people’s souls remain with loved ones after death				
Yes vs. No	0.009*	1.552	1.117	2.157
Belief that people’s souls disappear after death				
Yes vs. No	1.3x10^-4^**	1.742	1.310	2.317

Variables included in the regression analysis: age, sex, education, marital status, frequency of internet usage, history of psychiatric outpatient clinic visits, symptoms of depression, symptoms of anxiety or other psychiatric diagnosis, history of attempted suicide, belief that people’s souls go to heaven after death, belief that people’s souls go to hell after death, belief that people’s souls remain in the world after death, belief that people enter the cycle of reincarnation after death, belief that people repeat the dying process after death, belief that people’s souls remain with loved ones after death, belief that people’s souls disappear after death, uncertainty regarding where people’s souls go after death; only variables with statistical significance were shown in the Table.

*p < 0.05

**p < 0.001.

Notably, 90.3% of participants who completed the survey considered charcoal burning to be an easily accessible suicide method. Approximately 60% of participants perceived people who died by or attempted suicide through charcoal burning as people who lived alone, were accustomed to watching the news, and suffered from financial stress. Over half of those surveyed considered charcoal-burning suicide to be painless and a suicide method which leaves an intact corpse after death. Those who had considered suicide by charcoal burning themselves were more likely to perceive those who die by charcoal-burning suicide as individuals who are educated, use the Internet, and watch the news. Compared with participants who had not considered charcoal-burning suicide, those who had were more likely to romanticise it and considered it to be a method that is painless, leaves behind an ‘intact corpse’ (dead body with no missing parts), and beautiful (91.4% vs. 50.7%; 77.6% vs. 49.8%; and 50% vs. 19.5%; respectively; Table 2).

Logistic regression revealed that among those who completed the survey, a history of psychiatric outpatient visits, a history of attempted suicide, and perception of the ‘painlessness’ of charcoal-burning suicide predicted presence of ideation of charcoal-burning suicide (Table 4). The sensitivity analysis revealed that among those who had suicide ideation (n = 410), the perception of charcoal-burning suicide as ‘painless’ was the only significant independent factor (associated with an over five-fold increase) influencing the choice of charcoal burning (OR = 5.15; p = 0.003; 95% CI: 1.744–15.207) over other lethal methods after controlling for age, sex, education level, marital status, frequency of Internet usage, history of psychiatric symptoms or treatment, and conceptualisation of death or life after death (S1 Table).

**Table 4:** Odds ratios and 95% confidence intervals for the risk of having charcoal-burning suicide ideation (N = 1,343).

			95% CI for OR	
	Significance	OR	Lower	Upper
History of psychiatric outpatient clinic visits				
Yes vs. No	0.041*	2.527	1.037	6.158
History of attempted suicide				
Yes vs. No	0.010*	2.996	1.299	6.913
Do you think that charcoal-burning suicide is painless?				
Yes vs. No	1.6x10^-4^**	7.394	2.614	20.912

Variables included in the regression analysis: age, sex, education, marital status, frequency of internet usage, history of psychiatric outpatient clinic visits, symptoms of depression, symptoms of anxiety or other psychiatric diagnosis, history of attempted suicide, belief that people’s souls go to heaven after death, belief that people’s souls go to hell after death, belief that people’s souls remain in the world after death, belief that people enter the cycle of reincarnation after death, belief that people repeat the dying process after death, belief that people’s souls remain with loved ones after death, belief that people’s souls disappear after death, uncertainty regarding where people’s souls go after death, "Do you think that people who choose charcoal burning for suicide are accustomed to surfing the Internet?", "Do you think that people who choose charcoal burning for suicide are accustomed to watching the news?", "Do you think that people who choose charcoal burning for suicide are in debt?", "Do you think that people who choose charcoal burning for suicide live alone?", "Do you think that people who choose charcoal burning for suicide are poor?", "Do you think that people who choose charcoal burning for suicide are educated?", "Do you think that charcoal-burning suicide is painless?", "Do you think that charcoal-burning suicide keeps the body intact?", "Do you think that charcoal-burning suicide involves suffocation?", "Do you think that charcoal-burning suicide is beautiful?", "Do you think that charcoal is easily accessible?", "Do you believe that charcoal-burning suicide leads to burns or amputation?", "Do you believe that charcoal-burning suicide leads to lung injury?", "Do you believe that charcoal-burning suicide leads to delayed brain injury?"; only variables with statistical significance were shown in the Table.

*p < 0.05

**p < 0.001.

## Discussion

In the current study, we discovered that charcoal-burning suicide is perceived as easily accessible, painless, and a method that leaves behind an intact corpse. The survey respondents tended to portray those who died by or attempted charcoal-burning suicide as educated, frequent Internet users, and accustomed to watching the news. Furthermore, people who considered suicide through charcoal burning themselves tended to romanticise this suicide method as ‘beautiful’. ‘Painlessness’ may be the perception observed in this study which distinguished charcoal burning from other lethal methods among those contemplating suicide.

Charcoal-burning suicide is often misunderstood by the public, which may be partly attributable to the media implicitly conveying the message that charcoal burning is an easily accessible and painless means of suicide [9]. Over 90% of survey respondents in the current study believed that charcoal burning is easily accessible, and 52.5% perceived charcoal-burning suicide as ‘painless’. Notably, for those who considered suicide by charcoal burning themselves, 91.4% believed that charcoal-burning suicide is ‘painless’. Table 4 showed that among all survey respondents, the perceived ‘painlessness’ of charcoal-burning suicide was associated with an over seven-fold increased risk of choosing charcoal burning as a lethal method (OR = 7.394; p < 0.001; 95% CI: 2.614–20.912). Additionally, sensitivity analysis suggested that the perceived ‘painlessness’ was associated with an approximately five-fold increased risk of contemplating charcoal-burning suicide over other lethal means for those with suicidal ideation (OR = 5.15; p = 0.003; 95% CI: 1.744–15.207). The perceived ‘painlessness’ may be one of the most crucial perceptions of the public that has at least partly contributed to the surge of charcoal-burning suicides in susceptible societies.

Together with the perceived ‘painlessness’, the identified sociocultural perceptions towards charcoal-burning suicide in this study can be better understood in the construct of ‘capability for suicide’—fearlessness about death, practical capability, and pain tolerance—which researchers propose to facilitate the progression from suicidal ideations to attempts [19]. Up to 77.6% of respondents who contemplated charcoal-burning suicide perceived that this method leaves behind an intact body after death, whereas only 49.8% of those not contemplating charcoal-burning suicide had this perception. In Confucian thoughts, injuring one’s body—from a single hair to a bit of skin—is regarded as ‘nonfilial piety’. Further, the preservation of a complete corpse for burial is emphasised in traditional Chinese culture to ensure a good beginning for the next incarnation. Therefore, charcoal burning, which does not outwardly harm the body, may be preferable to other suicide methods that lead to more severe physical injury. The identified sociocultural perceptions towards charcoal burning—painlessness, barrier-free access, and intact body after death—correspond to pain tolerance, practical capability, and fearlessness about death, respectively, in the construct of ‘capability for suicide’. Had it been true that distinct features of charcoal burning provide ‘capability for suicide’ to a group of individuals who would have not attempted suicide if charcoal burning were unavailable, restricting access to this lethal method would prove its effectiveness. Although prior research showed that Taiwanese people using charcoal burning in the index suicide attempt had a higher risk of subsequent suicide death [20], those who attempted charcoal-burning suicide tended to use the same method on the next attempts [21] and they may be less prone to using other suicide methods. In this regard, restricting access to charcoal burning can reduce the related suicides as reported from prior efforts [22, 23], such as by prohibiting charcoal sales in convenience stores or establishing carbon monoxide detectors in certain areas.

Despite ‘fearlessness about death’ being a key component of ‘capability for suicide’, there is little information in the literature about the nature of fearlessness and its relationship with suicidal behaviours. In a preliminary analysis of a group of suicidal adolescents, fearlessness about death was a significant independent predictor of attempt, even when controlling for other commonly cited risk factors [24]. In the current study, we noted that conceptualisation of life after death such as the belief that the spirit remains with loved ones after death (OR = 1.552; p = 0.009; 95% CI: 1.117–2.157) or the spirit disappears after death (OR: 1.742; p < 0.001; 95% CI: 1.310–2.317) was associated with a greater risk of suicidal ideation (Table 3). Although speculative, this kind of conceptualisation of death or life after death can be strongly related to fearlessness about death. Besides, the beliefs such as the one that the spirit remains with loved ones after death are not religious but rather beliefs influenced by a popular culture that romanticises life after death. In a modern society in which socioeconomic, technological, and cultural forces rapidly shape people’s experiences, the impact of media and popular culture on people’s beliefs and suicidal behaviours can be more influential than before. Future studies are warranted to explore the complex interrelationships between different kinds of conceptualisation of death, fearlessness about death, and suicidal behaviours.

The influence of media on suicide is best illustrated when a celebrity suicide is reported. Both suicide deaths and calls to Lifeline may increase immediately [25]. In the current study, more than 60% of survey respondents considered those who watched the news to be more likely to choose charcoal burning as a suicide method. Most survey respondents perceived people who died by or attempted charcoal-burning suicide as living alone and suffering from financial stress, which are the features previously emphasised in the media reports [14]. The respondents tended to perceive those attempting charcoal-burning suicide as being educated and frequent Internet users. Indeed, a prior study reported that every 10% increase in Google searches was associated with a 4.3% increase in charcoal-burning suicide incidence in the same week whereas non-charcoal-burning suicide was not associated with Google search volume [26]. In addition, the survey respondents who had considered suicide by charcoal burning themselves were more likely than those who had not to romanticise charcoal burning as ‘beautiful’ (50% vs.19.5%). Despite the warning that media accounts romanticising suicide deaths may result in a surge of suicides [27–29], the media seldom follows guidelines for reporting suicide [30]. It is suggested that suicide prevention experts and media professionals work together to minimise the negative impacts of reports on suicide and to encourage help-seeking, particularly when the suicide incident is likely to receive extensive media coverage [25, 31]. The prevention of suicide by charcoal burning should also include monitoring and regulating online information that provides details of the methods and encouraging Internet service providers to provide psychoeducational and help-seeking information [26, 31].

Additionally, 7.1% of the survey respondents reported that they had sought any professional help owing to psychiatric symptoms or mental illnesses during their lifetimes. With regards to help seeking behaviours, stigmatisation of mental illness is recognised as a serious concern [32, 33]. For those with mental illness, stigmatisation may contribute to a high fatality rate, with public stigma and a higher level of internalised stigma exhibiting a correlation with suicidal behaviours [34, 35]. Moreover, stigmatisation and discrimination can be more devastating in the family-oriented Asian cultures as the stigma of mental illness influences the entire family [36]. Further interventions are thus needed to reduce stigmatisation, facilitate utilisation of mental health services, and probably reduce suicidal behaviours.

The strengths of the present study include examining the features of cultural preparedness and perceptions of charcoal-burning suicide which may be key to understanding the determining factors for the adoption of a new suicide method in susceptible communities. Although exploratory in nature, the present study also represented a rare attempt to investigate the potential associations between personal belief in life after death and suicidal behaviours. Our study has several limitations. We recruited participants online and therefore, they may be more representative of a population that is younger and has more frequent Internet use, which limits the generalisability of the results. Despite the fact that only 12.1% of the participants reported that they had suffered from psychiatric symptoms including depression, anxiety, or sleep disturbance during their lifetime, it remains possible that individuals interested in mental health or suffering from psychiatric symptoms may be more likely to complete the survey. Given the number of covariates used in the regression models, multicollinearity can inflate the variance of the parameter estimates which should be kept in mind when interpreting the current results. To assess the on-line survey quality as presented in this study, future efforts to conduct an additional survey with an independent sample via individual interview may be warranted.

In this exploratory study to understand the decisions of charcoal-burning suicide, our results showed that charcoal-burning suicide is perceived as easily accessible, painless, and a method that leaves behind an intact corpse. When put in the context of ‘capability for suicide’—fearlessness about death, practical capability, and pain tolerance, the identified sociocultural perceptions bring insights on the plausible explanation for the rapid adoption of charcoal burning as a leading suicide method in Taiwan. Future efforts for the prevention of suicide by charcoal burning should be focussing on the modification of the related perceptions, restricting access to charcoal burning, as well as monitoring and regulating online information that provides details of the method.

## Supporting information

S1 Table Odds ratios and 95% confidence intervals for the risk of having charcoal-burning suicide ideation among those contemplating suicide, n = 410 (sensitivity analysis). Variables included in the regression analysis: age, sex, education, marital status, frequency of internet usage, history of psychiatric outpatient clinic visits, symptoms of depression, symptoms of anxiety or other psychiatric diagnosis, history of attempted suicide, belief that people’s souls go to heaven after death, belief that people’s souls go to hell after death, belief that people’s souls remain in the world after death, belief that people enter the cycle of reincarnation after death, belief that people repeat the dying process after death, belief that people’s souls remain with loved ones after death, belief that people’s souls disappear after death, uncertainty regarding where people’s souls go after death, "Do you think that people who choose charcoal burning for suicide are accustomed to surfing the Internet?", "Do you think that people who choose charcoal burning for suicide are accustomed to watching the news?", "Do you think that people who choose charcoal burning for suicide are in debt?", "Do you think that people who choose charcoal burning for suicide live alone?", "Do you think that people who choose charcoal burning for suicide are poor?", "Do you think that people who choose charcoal burning for suicide are educated?", "Do you think that charcoal-burning suicide is painless?", "Do you think that charcoal-burning suicide keeps the body intact?", "Do you think that charcoal-burning suicide involves suffocation?", "Do you think that charcoal-burning suicide is beautiful?", "Do you think that charcoal is easily accessible?", "Do you believe that charcoal-burning suicide leads to burns or amputation?", "Do you believe that charcoal-burning suicide leads to lung injury?", "Do you believe that charcoal-burning suicide leads to delayed brain injury?"; only variables with statistical significance were shown in the Table. *p < 0.05.
(DOCX)
