# Supplementary material for: Perceptions towards charcoal-burning suicide and the surge of this lethal method in Taiwan
Source: PLoS One. 2022 Jan 21;17(1):e0262384. doi: 10.1371/journal.pone.0262384 (PMC8782296; doi:10.1371/journal.pone.0262384)
Supplement: S1 Table — Variables included in the regression analysis: age, sex, education, marital status, frequency of internet usage, history of psychiatric outpatient clinic visits, symptoms of depression, symptoms of anxiety or other psychiatric diagnosis, history of attempted suicide, belief that people’s souls go to heaven after death, belief that people’s souls go to hell after death, belief that people’s souls remain in the world after death, belief that people enter the cycle of reincarnation after death, belief that people repeat the dying process after death, belief that people’s souls remain with loved ones after death, belief that people’s souls disappear after death, uncertainty regarding where people’s souls go after death, "Do you think that people who choose charcoal burning for suicide are accustomed to surfing the Internet?", "Do you think that people who choose charcoal burning for suicide are accustomed to watching the news?", "Do you think that people who choose charcoal burning for suicide are in debt?", "Do you think that people who choose charcoal burning for suicide live alone?", "Do you think that people who choose charcoal burning for suicide are poor?", "Do you think that people who choose charcoal burning for suicide are educated?", "Do you think that charcoal-burning suicide is painless?", "Do you think that charcoal-burning suicide keeps the body intact?", "Do you think that charcoal-burning suicide involves suffocation?", "Do you think that charcoal-burning suicide is beautiful?", "Do you think that charcoal is easily accessible?", "Do you believe that charcoal-burning suicide leads to burns or amputation?", "Do you believe that charcoal-burning suicide leads to lung injury?", "Do you believe that charcoal-burning suicide leads to delayed brain injury?"; only variables with statistical significance were shown in the Table. *p < 0.05. (DOCX) [file pone.0262384.s001.docx]

**S1 Table**

*Odds ratios and 95% confidence intervals for the risk of having charcoal-burning suicide ideation among those contemplating suicide, n = 410 (sensitivity analysis)*

|  |  |  |  | 95% CI for OR | |
| --- | --- | --- | --- | --- | --- |
|  |  | Significance | OR | Lower | Upper |
| Do you think that charcoal-burning suicide is painless? | | | | |  |
| Yes vs. No |  | 0.003* | 5.150 | 1.744 | 15.207 |
| Variables included in the regression analysis: age, sex, education, marital status, frequency of internet usage, history of psychiatric outpatient clinic visits, symptoms of depression, symptoms of anxiety or other psychiatric diagnosis, history of attempted suicide, belief that people’s souls go to heaven after death, belief that people’s souls go to hell after death, belief that people’s souls remain in the world after death, belief that people enter the cycle of reincarnation after death, belief that people repeat the dying process after death, belief that people’s souls remain with loved ones after death, belief that people’s souls disappear after death, uncertainty regarding where people’s souls go after death, "Do you think that people who choose charcoal burning for suicide are accustomed to surfing the Internet?", "Do you think that people who choose charcoal burning for suicide are accustomed to watching the news?", "Do you think that people who choose charcoal burning for suicide are in debt?", "Do you think that people who choose charcoal burning for suicide live alone?", "Do you think that people who choose charcoal burning for suicide are poor?", "Do you think that people who choose charcoal burning for suicide are educated?", "Do you think that charcoal-burning suicide is painless?", "Do you think that charcoal-burning suicide keeps the body intact?", "Do you think that charcoal-burning suicide involves suffocation?", "Do you think that charcoal-burning suicide is beautiful?", "Do you think that charcoal is easily accessible?", "Do you believe that charcoal-burning suicide leads to burns or amputation?", "Do you believe that charcoal-burning suicide leads to lung injury?", "Do you believe that charcoal-burning suicide leads to delayed brain injury?"; only variables with statistical significance were shown in the Table.  *p < 0.05. | | | | | |
